# Clinical Relevance of *CDH1* and *CDH13* DNA-Methylation in Serum of Cervical Cancer Patients

**DOI:** 10.3390/ijms13078353

**Published:** 2012-07-05

**Authors:** Abida Abudukadeer, Rania Bakry, Georg Goebel, Irene Mutz-Dehbalaie, Andreas Widschwendter, Günther K. Bonn, Heidi Fiegl

**Affiliations:** 1 Department of Gynecology, The First Affiliated Hospital, Medical University of Xinjiang, Urumqi 830000, China; E-Mail: abida0724@126.com; 2 Institute of Analytical Chemistry and Radiochemistry, Leopold-Franzens University, Innsbruck, Tirol 6020, Austria; E-Mails: rania.bakry@uibk.ac.at (R.B.); guenther.bonn@uibk.ac.at (G.K.B.); 3 Department of Medical Statistics, Informatics and Health Economics, Innsbruck Medical University, Innsbruck, Tirol 6020, Austria; E-Mail: georg.goebel@i-med.ac.at; 4 Department of Gynecology and Obstetrics, Innsbruck Medical University, Innsbruck, Tirol 6020, Austria; E-Mails: irene.mutz-dehbalaie@i-med.ac.at (I.M.-D.); andreas.widschwendter@i-med.ac.at (A.W.)

**Keywords:** cancer biomarker, epigenomics, DNA-methylation, prognosis, translational cancer research

## Abstract

This study was designed to investigate the DNA-methylation status of *E*-cadherin (*CDH1*) and *H*-cadherin (*CDH13*) in serum samples of cervical cancer patients and control patients with no malignant diseases and to evaluate the clinical utility of these markers. DNA-methylation status of *CDH1* and *CDH13* was analyzed by means of MethyLight-technology in serum samples from 49 cervical cancer patients and 40 patients with diseases other than cancer. To compare this methylation analysis with another technique, we analyzed the samples with a denaturing high performance liquid chromatography (DHPLC) PCR-method. The specificity and sensitivity of *CDH1* DNA-methylation measured by MethyLight was 75% and 55%, and for *CDH13* DNA-methylation 95% and 10%. We identified a specificity of 92.5% and a sensitivity of only 27% for the *CDH1* DHPLC-PCR analysis. Multivariate analysis showed that serum *CDH1* methylation-positive patients had a 7.8-fold risk for death (95% CI: 2.2–27.7; *p* = 0.001) and a 92.8-fold risk for relapse (95% CI: 3.9–2207.1; *p* = 0.005). We concluded that the serological detection of *CDH1* and *CDH13* DNA-hypermethylation is not an ideal diagnostic tool due to low diagnostic specificity and sensitivity. However, it was validated that *CDH1* methylation analysis in serum samples may be of potential use as a prognostic marker for cervical cancer patients.

## 1. Introduction

Cervical cancer continues to be one of the leading female genital cancers worldwide. It is a common cancer and a serious threat to women’s lives in developing countries, where about 80% of all cases occur. Although infection with high-risk human papilloma viruses (HPV) is the main etiological factor in the development of cervical cancer, the majority of patients with HPV-associated lesions do not progress to invasive cancer. Other factors are therefore also involved in cervical carcinogenesis. Promoter DNA-hypermethylation has been shown to play an important role in cervical carcinogenesis [1–4]. Hypermethylation of CpG islands in the promoter region of genes is an important marker of gene inactivation and a cutting edge research area in the epigenetic study of tumor biomarkers. Numerous studies have demonstrated tumor specific alterations such as aberrant DNA-methylation in plasma or serum [5–9].

Cadherins are transmembrane glycoproteins expressed on the epithelial cell surface that mediate intercellular Ca^2+^-dependent adhesion, which is important for maintaining normal tissue structure [10]. Reduced expression of cadherin family members such as *E*-cadherin (*CDH1*) and *H*-cadherin (*CDH13*) is observed in many tumors [11,12]. While both genes are accepted as tumor-suppressor genes, the molecular mechanism is not yet clear.

Recent studies have indicated that DNA-methylation of *CDH1* and *CDH13* may lead to decreased *E*-cadherin and *H*-cadherin expression [12,13]. It is also known that decreasing *E*-cadherin expression contributes to enhanced metastasizing activity or more aggressive malignant tumors [14]. Although some advances in surgery, chemotherapy, and radiotherapy have been made recently, the high mortality rate is related in part to the high proportion of patients already suffering from advanced disease at the time of their first diagnosis. The identification of biomarkers for early detection of cervical cancer and for prognosis and prediction is therefore a very important task for the prevention and treatment of cervical cancer. Recently, we identified *CDH1* and *CDH13* DNA-methylation in serum samples taken at the time of diagnosis as an independent prognostic marker in cervical cancer patients with no concurrent chemo- or radiation therapy [15].

In the present study, we investigated the DNA-methylation status of *CDH1* and *CDH13* in serum samples from 49 cervical cancer patients and 40 patients with benign diseases. We used MethyLight PCR to analyze specificity and sensitivity and to evaluate the prognostic significance of these DNA-methylation-based markers. Finally, we compared the MethyLight method with the denaturing high-performance liquid chromatography (DHPLC) of methylation-specific PCR products for *CDH1* and *CDH13*.

## 2. Results and Discussion

### 2.1. Specificity and Sensitivity of *CDH1* and *CDH13* DNA-Methylation in Serum Samples

To determine whether the DNA-methylation status of *CDH1* or *CDH13* in serum samples has a diagnostic value for cervical cancer, we used MethyLight analysis to investigate the frequency of DNA-methylation of *CDH1* and *CDH13* in serum samples from 40 patients with non-malignant diseases and 49 cervical cancer patients. In non-malignant serum samples, aberrant DNA-methylation for *CDH1* was present in 10 of 40 serum samples (75% specificity). Among the patients with invasive cervical cancer, the DNA-methylation frequency for *CDH1* was 55% (27 of 49; 55% sensitivity). For *CDH13*, we identified in non-malignant serum samples aberrant DNA-methylation in two of 40 serum samples (95% specificity), but in only five of 49 serum samples from patients with invasive cervical cancer (sensitivity 10%).

A statistically significant higher frequency of DNA-methylation for *CDH1*, but not for *CDH13*, was observed in cervical cancer patients in comparison to patients with benign diseases (*p* = 0.004).

### 2.2. Correlation of *CDH1* and *CDH13* DNA-Methylation and Survival

To determine whether any prognostic significance was connected to differences in the detection of *CDH1* or *CDH13* DNA-methylation in serum samples of cervical cancer patients, we compared the clinical outcomes of cervical cancer patients with and without *CDH1* or *CDH13* DNA-methylation. Neither *CDH1* nor *CDH13* DNA-methylation correlated with clinicopathological features (Table 1).

#### 2.2.1. Univariate Survival Analysis

**Figure 1:**
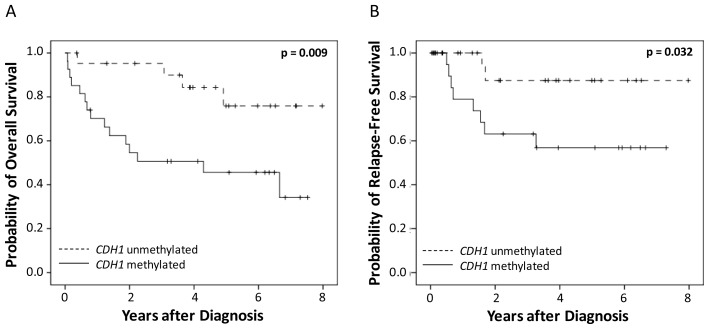
Kaplan Meier survival analysis in serum samples from 49 cervical cancer patients. *CDH1* DNA-methylation measured by MethyLight PCR and (**A**) overall survival and (**B**) relapse-free survival.

Univariate survival analysis revealed that the detection of methylated *CDH1* DNA in serum samples was significantly associated with poor outcome for overall and relapse free survival. This was determined by means of MethyLight PCR (*p* = 0.009 and *p* = 0.032, respectively; Table 2, Figure 1). A combination of *CDH1* and *CDH13* DNA-methylation data did not improve the analysis (data not shown).

#### 2.2.2. Multivariate Survival Analysis

To assess independent prognostic significance, a Cox proportional hazard model analysis was carried out including age, tumor stage, grade of differentiation, chemotherapy and *CDH1* and *CDH13* methylation status in serum. Serum *CDH1* methylation-positive patients had a 7.8-fold risk for death (95% CI: 2.2–27.7; *p* = 0.001) and a 92.8-fold risk for relapse (95% CI: 3.9–2207.1; *p* = 0.005) in comparison to *CDH1* methylation-negative patients (Table 3).

### 2.3. Comparison of MethyLight PCR and DHPLC-PCR

Furthermore, we compared the *CDH1* results of the MethyLight assay with the results of a DHPLC PCR in all 89 serum samples. We found only a modest correlation between these data (*r* = 0.496; *p* < 0.001). Only 15 of all 37 (41%) MethyLight positive samples could also be detected by means of DHPLC PCR, whereas 15 of the 16 (94%) DHPLC positive samples were detected by MethyLight PCR.

Moreover, the DHPLC method showed a statistically significant higher frequency of DNA-methylation for *CDH1* in cervical cancer patients than in patients with benign diseases (*p* = 0.020). Among the patients with invasive cervical cancer, the DNA-methylation frequency for *CDH1* was only about 27% (13 of 49). In non-malignant serum samples, we detected aberrant DNA-methylation in three of 40 serum samples (7.5%). Consequently, we identified a specificity of 92.5% and a sensitivity of only 27% for the *CDH1* DHPLC PCR analysis. With this method we were not able to identify a statistically significant difference between cervical cancer patients and patients with benign diseases.

### 2.4. Discussion

Cervical cancer is a leading cause of cancer-related death in women. Several clinical and histopathological characteristics, *i.e.*, tumor stage, lymph node status, and vascular invasion, have been shown to be prognostic factors for recurrent disease [16,17]. New objective diagnostic, prognostic and predictive biomarkers for cervical cancer are needed.

Recently we identified *CDH1* and *CDH13* DNA-methylation in serum samples taken at the time of diagnosis as an independent prognostic marker in cervical cancer patients with no concurrent chemo- or radiation therapy [15].

In the present study, the methylation status of *CDH1* and *CDH13* genes in serum samples from 49 cervical cancer patients who were treated according to the guidelines and 40 patients with diseases other than cancer was reevaluated using MethyLight assay technology. The DNA-methylation frequency for *CDH1* was 55% in the serum samples from patients with invasive cervical cancer. In our previous study, we identified a frequency of 42% in serum [15]. Other groups describe frequencies of about 51.1–80.5% in cervical cancer tissue samples but not in serum samples. [18,19]. For *CDH13* methylation, a frequency of only 10% was observed in serum samples from cervical cancer patients. Distribution of *CDH1* but not *CDH13* methylation within the cervical cancer and non-cancer group showed statistically significant differences. For the detection of cervical cancer based on the *CDH1* methylation analysis in serum samples, however, the specificity and sensitivity values of 75% and 55% are not convincing. Therefore the serological detection of *CDH1* and *CDH13* DNA-methylation in serum does not appear to be a suitable method for cervical cancer detection. There is currently no methylation marker that can be readily translated for use in cervical cancer screening or triage settings [20].

To determine whether the methylation status of *CDH1* and *CDH13* in serum samples has also a prognostic value for cervical cancer, we compared serum DNA-methylation of these genes with the overall and relapse-free survival of patients. We found that there was a significant difference in the overall and the relapse-free survival for patients with methylated *CDH1* DNA in their pre-treatment serum samples. These findings are in accordance with our previously published study where only patients without concurrent chemo- or radiation therapies were analyzed [15]. In that study, we observed a higher *CDH1* DNA-methylation frequency in serum samples of patients with FIGO Stage III and no association with tumor grade. In the present study, we found only a non-statistically significant trend of a higher *CDH1* DNA-methylation frequency in serum samples of patients with FIGO IV and again no association with tumor grade. The low number of patients in the present study may explain the slight difference compared to the previous study. In our present study the combination of *CDH1* and *CDH13* DNA-methylation data did not improve the survival results. However, further studies with larger numbers of patients are required to confirm our findings.

Finally, we compared MethyLight technology and DHPLC-PCR for *CDH1* DNA-methylation analysis. Using DHPLC-PCR, we detected only 41% of all MethyLight positive samples, but the majority (94%) of the DHPLC positive samples was also detected by MethyLight PCR.

The comparison of methods showed a higher detection rate by MethyLight PCR. Using DHPLC-PCR for *CDH1,* it was not possible to distinguish statistically significantly between patients with cervical cancer and patients with benign diseases. The high sensitivity of MethyLight PCR technology could be a reason for the discrepancy in the methylation frequencies detected by MethyLight and DPHLC. MethyLight PCR can detect a single methylated allele in 10^5^ unmethylated alleles [21]. Previously, Eads et al. showed that MethyLight PCR is at least 10 times more sensitive than reported for the highly sensitive MSP technology [21].

The findings described in this manuscript suggest that the more labor-intensive DHPLC-PCR is not an ideal method for *CDH1* methylation analysis in serum samples.

## 3. Experimental Section

### 3.1. Patients

For this retrospective study we analyzed serum samples from 49 cervical cancer patients (ages 31.4–93.4 years; median age, 60.5 years) and 40 patients with non-malignant gynecological diseases (ages 30.7–85.8 years; median age 56.7). All patients were treated at the Department of Obstetrics and Gynecology, Innsbruck Medical University, Austria. Treatment was conducted according to international standards. All patients were monitored within the department’s outpatient follow-up program. The median observation period of the cervical-cancer patients was 2.2 years (1 month to 8.0 years). Overall, 24% of the patients had surgery (*n* = 12), 53% received chemotherapy (*n* = 26) and 78% received radiotherapy (*n* = 38). Radiation therapy was applied in combination with chemotherapy in 49% (*n* = 24) of patients and 18% of patients received no adjuvant therapy (*n* = 9). The study was approved by the ethics committee of the Innsbruck Medical University (reference number: AN3568) and conducted in accordance with the Declaration of Helsinki.

### 3.2. Serum Characteristics

All serum samples were taken on the date of diagnosis and before initial treatment. Serum samples were stored at −50 °C until analysis was performed. The median storage age of the serum samples from cervical cancer patients was 4.4 years (5.5 months to 5.8 years), and 4.9 years (1.2–5.9 years) for patients with non-malignant gynecological diseases.

### 3.3. DNA Isolation and MethyLight PCR Analysis

DNA from 1 mL serum samples was isolated using the ChargeSwitch gDNA kit from Invitrogen (Life technologies, Paisley, UK) according to the manufacturer’s protocol. Sodium bisulfite conversion of genomic DNA was performed using the EZ DNA Methylation-Gold KitTM (Zymo Research, Orange, CA, USA) according to the manufacturer’s recommendations. Sodium bisulfite-treated genomic DNA was analyzed by means of the MethyLight method, a fluorescence-based, real-time PCR assay, as previously described [15,21]. Two sets of primers and probes, designed specifically for bisulfite-converted DNA, were used: a methylated set for the gene of interest and a reference set, collagen (COL2A1), to normalize for input DNA. Specificity of the reactions for methylated DNA was confirmed separately using SssI-treated (New England Biolabs, Beverly, MA, USA) human white blood cell DNA, which is heavily methylated. The percentage of fully methylated molecules at a specific locus was calculated by dividing the GENE:COL2A1 ratio of a sample by the GENE:COL2A1 ratio of SssI-treated white blood cell DNA and multiplying by 100. The abbreviation PMR (percentage of fully methylated reference) indicates this measurement. For each MethyLight reaction, 10 μL of bisulfite-treated genomic DNA were used. A gene was deemed unmethylated if the PMR value was 0. To verify the reproducibility of each assay, the normalized value (GENE:COL2A1) of the standard sample was checked for all PCR runs. The primers and probes used for MethyLight reactions have been described recently [15].

### 3.4. Denaturing High-Performance Liquid Chromatography Analysis (DHPLC) PCR

The primers used in PCR for DHPLC were determined with the assistance of the software Methyl Primer Express v1.0 (Applied Biosystems, Foster City, CA, USA). The primers are designed to amplify 403 bp (*CDH1*) and 348 bp (*CDH13*) of bisulfate-modified DNA, irrespective of methylation status. *CDH1* Forward primer: 5′-TTTTAGTTTGGGTGAAAGAGT-3′; *CDH1* Reverse Primer: 5′-AACTCACAAATACTTTACAATTCC-3′; *CDH13* Forward Primer: 5′-TTTGTTTTAGGTAGGGAAGAGG-3′; *CDH13* Reverse Primer: 5′-AAAACCAAAATTACCCCACTTA- 3′.

PCR reactions were performed in a final volume of 25 μL containing 1 U of HotStarTaq DNA Polymerase (Qiagen, Hilden, Germany), 0.2 μM dNTP mix (Qiagen, Hilden, Germany), 250 nM of each primer, 1× buffer and 10 μL of bisulfite modified DNA. All reactions were performed under the following thermal cycling conditions: 95 °C for 15 min, followed by 35 cycles of 94 °C for 1 min, 50 °C for 30 sec, 72 °C for 1 min, and a final extension step at 72 °C for 10 min. After amplification, PCR products were visualized by gel electrophoresis.

Afterwards, PCR products were analyzed directly by the Transgenomic WAVE DHPLC instrument (San Jose, CA, USA) and a DNASep ™ 50 × 4.6 mm i.d. cartridge was used as a column (Transgenomic, Omaha, NE, USA). Separation of products was conducted at 54.3 °C and 57.6 °C for *CDH1* and *CDH13*, respectively, by continuously mixing buffer B (0.1 M triethylammonium acetate, 25% acetonitrile) with buffer A (0.1 M triethylammonium acetate), either over 5 min: 57–66% (*CDH1*) or 50–75% (*CDH13*) at 0.9 mL per minute flow rate. The partially denaturing temperature at which samples were run was determined for each assay using Wavemaker software version 4.1.40 (Transgenomic, Omaha, NE, USA). The average partially denaturing temperature of the methylated amplicon was used to separate the target fragments with various degrees of methylation.

### 3.5. Statistical Analysis

Associations between categorical variables were tested with Pearson’s Chi square test. The Kaplan-Meier method was used for univariate survival analysis, and the log rank test was used to assess the difference between groups. A Cox proportional hazards model applying a backward variable selection procedure based on the likelihood-ratio test was used for multivariate survival analysis. The correlation between the MethyLight PCR data and the DHPLC-PCR data was analyzed using the Pearson’s correlation coefficient. A *p* value of <0.05 was considered statistically significant. These statistical calculations were performed using SPSS, version 13.0.

## 4. Conclusions

From the findings of this study, we conclude that serological detection of *CDH1* or *CDH13* promoter hypermethylation is not able to predict invasive cervical cancers. However, it was shown that *CDH1* DNA-methylation analysis may be of potential use as a prognostic marker for cervical cancer patients.

## Figures and Tables

**Table 1:** Clinicopathological features and *CDH1* and *CDH13* DNA-methylation in serum samples of 49 cervical cancer patients.

	MethyLight DHPLC PCR			MethyLight	
		
Characteristics	No. of Cases (*n* = 49)	*CDH1* (%) *	*CDH13* (%) *	No. of Cases (*n* = 49)	*CDH1* (%) *
**Stage**					
FIGO I	19	10 (53%)	3 (16%)	5 (26%)	3 (16%)
FIGO II	13	8 (62%)	0 (0%)	3 (23%)	4 (31%)
FIGO III	12	5 (42%)	1 (8%)	3 (25%)	0 (0%)
FIGO IV	5	4 (80%)	1 (20%)	2 (40%)	0 (0%)
**Tumor grade**					
I	6	3 (50%)	0 (0%)	1 (17%)	0 (0%)
II	31	18 (58%)	5 (16%)	9 (29%)	5 (16%)
III	12	6 (50%)	0 (0%)	3 (25%)	2 (17%)
**Histology**					
Squamous cell carcinoma	41	21 (51%)	4 (10%)	9 (22%)	4 (10%)
Small cell carcinoma	8	6 (75%)	1 (13%)	4 (50%)	3 (38%)
**Age (years)**					
<50	11	5 (45%)	0 (0%)	4 (36%)	2 (18%)
≥50	38	22 (58%)	5 (13%)	9 (24%)	5 (13%)

* Pearson’s Chi square test showed no significant associations with clinicopathological features.

**Table 2:** Univariate survival analysis. Overall and relapse-free survival in 49 patients with primary cervical cancer.

	Overall survival		Relapse-free survival	
	
Variables	No. patients	*p* value	No. patients	*p* value
	
	(died/total)	(logrank-test)	(relapsed/total)	(logrank-test)
**Age**				
<50	3/11	0.332	2/11	0.513
≥50	16/38		8/38	
**Stage**				
FIGO I	6/19	**<0.001**	2/19	**<0.001**
FIGO II	3/13		2/13	
FIGO III	5/12		5/12	
FIGO IV	5/5		1/5	
**Tumor grade**				
I	0/6	0.127	0/6	0.058
II	13/31		5/31	
III	6/12		5/12	
**Surgery**				
no	16/37	0.180	8/37	0.339
yes	3/12		2/12	
**Chemotherapy**				
no	13/23	**0.010**	4/23	0.991
yes	6/26		6/26	
**Radiation therapy**				
no	4/10	0.586	1/10	0.375
yes	14/38		9/38	
***CDH1*** **DNA-methylation**				
negative	4/22	**0.009**	2/22	**0.032**
positive	15/27		8/27	
***CDH13*** **DNA-methylation**				
negative	17/44	0.777	9/44	0.679
positive	2/5		1/5	

**Table 3:** Multivariate survival analysis. Overall and relapse-free survival in 49 patients with primary cervical cancer.

	Overall survival		Relapse-free survival	
	
	HR (95% CI)	*p* value	HR (95% CI)	*p* value
**Age**				
<50 *vs*. ≥50	- *	-	- *	-
**Stage**				
FIGO I/II *vs*. III/IV	6.4 (2.1–19.1)	0.001	24.6 (3.5–175.2)	0.001
**Tumor grade**				
I/II *vs.* III	- *	-	9.0 (1.5–54.1)	0.016
**Chemotherapy**				
no *vs*. yes	0.2 (0.1–0.5)	0.002	0.2 (0.03–1.6)	0.130
***CDH1*** **DNA-methylation**				
negative *vs.* positive	7.8 (2.2–27.7)	0.001	92.8 (3.9–2207.1)	0.005
***CDH13*** **DNA-methylation**				
negative *vs*. positive	2.3 (0.4–12.3)	0.3	- *	-

* Variable dropped in backward selection.
